# The Impact of Tetracycline on the Soil Microbiome and the Rhizosphere of Lettuce (*Lactuca sativa* L.)

**DOI:** 10.3390/ijms26072854

**Published:** 2025-03-21

**Authors:** Magdalena Krupka, Lidia Wolska, Lidia Piechowicz, Katarzyna Głowacka, Agnieszka I. Piotrowicz-Cieślak

**Affiliations:** 1 Department of Plant Physiology, Genetics and Biotechnology, University of Warmia and Mazury, 10-719 Olsztyn, Poland; magdalena.krupka@uwm.edu.pl (M.K.); katarzyna.glowacka@uwm.edu.pl (K.G.); 2 Department of Environmental Toxicology, Faculty of Health Sciences, Medical University of Gdansk, 80-204 Gdansk, Poland; lidia.wolska@gumed.edu.pl; 3 Department of Medical Microbiology, Faculty of Medicine, Medical University of Gdansk, 80-204 Gdansk, Poland; lidia.piechowicz@gumed.edu.pl

**Keywords:** bacteria, plants, soil, rhizosphere microbiome

## Abstract

The impact of tetracycline on the soil and rhizosphere microbiome of lettuce was analyzed. Soil was collected from an agricultural field regularly fertilized with manure, and tetracycline was added at two concentrations (5 mg/kg and 25 mg/kg). In untreated soil, dominant bacteria included *Proteobacteria* (43.17%), *Bacteroidota* (17.91%), and *Firmicutes* (3.06%). Tetracycline addition caused significant shifts in the microbiome composition, notably increasing *Actinobacteriota* (22%) and favoring *Mycobacterium tuberculosis* (low concentration) and *Mycobacterium holsaticum* (high concentration). *Proteobacteria* decreased by 21%, possibly indicating antibiotic resistance development. An increase in *Firmicutes*, particularly *Bacillales*, suggested a selection for resistant strains. In the lettuce rhizosphere, tetracycline-induced changes were less pronounced than in soil. *Proteobacteria* remained dominant, but taxa like *Burkholderiales* and *Chitinophagales* increased in response to tetracycline. The rise in chitin-degrading bacteria might result from fungal overgrowth linked to the bacteriostatic effects of tetracycline. Pathogens such as *M. tuberculosis*, observed in the soil, were not detected in the lettuce rhizosphere.

## 1. Introduction

Plants are a habitat for 10^4^–10^10^ bacteria per gram of plant material [1]. The largest groups of bacteria inhabiting plants belong to the phyla *Proteobacteria, Bacteroidetes*, *Firmicutes*, and *Actinobacteria* [2]. These bacteria colonize the roots (rhizosphere), leaves (phyllosphere), fruits (carposphere), and internal plant tissues (endosphere). While many bacterial species inhabiting plants are considered pathogens, the plant microbiome also plays a crucial role in plant health, growth, and stress resistance [3]. However, understanding the role and composition of microorganisms inhabiting plants is still far from complete [4], and the factors shaping the plant microbiome are not fully defined. Increasing research suggests that microorganisms inhabiting plant tissues can colonize the human body [5]. One of the significant factors affecting the human microbiome is diet [6]. Until now, most attention has been focused on microorganisms from fermented foods [1] and the presence of pathogens in animal-derived products [7]. The “One Health” concept has recently highlighted plants as a crucial link between microorganisms and the human body. However, studies exploring this connection remain limited, underscoring the need for further research to better understand the role of plant-associated microbiomes in the context of human and environmental health.

Wicaksono et al. [5] conducted the first study on the colonization of the gut microbiome by bacteria associated with vegetables and fruits, demonstrating that strains inhabiting fresh produce are detected in human intestines and form a functional part of the human microbiome. Similarly, the research presented here explores how tetracycline, even at environmentally detectable concentrations, affects the microbiome structure of both the soil and the rhizosphere of lettuce. Since the soil and rhizosphere are known sources of bacteria for the phyllosphere—the microbial community directly associated with edible plant surfaces—this study provides novel insights into how antibiotics in agricultural ecosystems might influence not only soil microbial dynamics but also the edible microbiome that can potentially interact with the human gut.

The largest groups of bacteria inhabiting plants, such as genera *Bacteroidetes* and *Firmicutes*, are dominant in the human intestine and play an important role in the process of digestion and energy absorption [8]. As the major dominants in core microbiota, they are the key regulators of body homeostasis, involving both intestinal and extra-intestinal effects by influencing many physiological functions, such as metabolism, the maintenance of barrier homeostasis, inflammation, and hematopoiesis [9]. Maintaining the right proportion of *Bacteroides* and *Firmicutes* strains is important, especially when it comes to combating obesity and related metabolic disorders. The literature suggests that a high *Firmicutes/Bacteroidetes* (F/B) ratio may be associated with a higher risk of metabolic syndrome, diabetes, and obesity [10]. Therefore, microbiological control of vegetables and fruits and understanding their microbiome composition during cultivation are crucial for ensuring food safety and protecting consumer health. Soil is the largest reservoir of environmental microorganisms and serves as the source of the microbiome for the plants grown in it [11]. The greatest threat to environmental microorganisms is antibiotics, which are still widely used in commercial livestock production. These compounds are used both prophylactically and therapeutically. By 2030, the estimated use of these pharmaceuticals in animal production will exceed 100,000 tons [12]. Only a small portion of antibiotics is effectively metabolized in animal bodies. Most of the administered dose is excreted directly by the animal through urine and feces [13]. Antibiotic residue concentrations in manure, sewage sludge, and soils vary widely, ranging from nanograms to milligrams per kilogram [14,15]. The concentration of tetracyclines varies considerably; for example, tetracycline content in fresh chicken manure ranges from 1.02 mg/kg [16] to 53.0 mg/kg in pig manure [17]. De La Torre et al. [18] reported that the average antibiotic levels range from 0.0008 to 2.7 mg/kg. Fang et al. [19] found that 56 types of antibiotics were detected in agricultural soils at concentrations ranging from undetectable to over 7 mg/kg, with the highest accumulation observed in vegetable fields and orchards. Antibiotics occurring at nonlethal and subinhibitory concentrations (typically nanograms per kilogram of soil) can have long-term indirect effects associated with the development of intrinsic or acquired antibiotic resistance in microorganisms [20,21,22]. Acquired resistance may result from the transfer of resistance genes from the environment and/or horizontal gene transfer, wherein genetic material (small DNA fragments) is transmitted from one bacterium to another, even if the latter is phylogenetically distant [14,23]. Antimicrobial resistance may also develop due to the presence of biocides and heavy metals through co-resistance, as antibiotic resistance genes (ARGs) and metal resistance genes are often located together on the same genetic element [24,25,26]. An increase in the number of antibiotic-resistant bacteria in the environment can enhance the transmission of resistance to organisms, potentially leading to human diseases.

Unprocessed waste containing antibiotic residues is then applied as an organic fertilizer to agricultural fields, introducing antibiotics into the soil. Most research on plant microbiomes focuses on manure as a source of antibiotics, microorganisms, and antibiotic-resistance genes (ARGs). In addition to manure, a significant source of antibiotics in agricultural soils is irrigation water, agricultural waste, sewage sludge, and spread from nearby livestock farms [27]. However, the direct impact of the veterinary antibiotics present in the soil on plant microbiota composition is often overlooked and only fragmentarily understood.

Plants actively recruit their microbiome from the surrounding environment, with microorganisms typically transported horizontally from the soil and rhizosphere through vascular tissues to the aerial parts of the plant [28]. Changes in the composition of microorganisms inhabiting the soil and rhizosphere can thus induce changes in the endosphere and phyllosphere of plants [29], negatively impacting plant health and raising concerns about the transfer of pathogens and ARGs to edible plant parts. The first step in studying plant microbiomes should, therefore, be the assessment of the composition and diversity of microorganisms inhabiting the soil and rhizosphere, particularly in plants growing in environments contaminated with antibiotics. This work represents a pioneering effort in this regard, specifically focusing on lettuce grown in soil with tetracycline contamination.

Tetracycline is one of the most commonly used veterinary antibiotics due to its broad spectrum of activity, low cost, and ease of administration [30]. Tetracycline antibiotic residues are detected in most agricultural soils [14].

The aim of this study was to analyze the impact of tetracycline on the microbiome structure of soil and the rhizosphere of lettuce (*Lactuca sativa* L.) grown under contamination with this antibiotic. The study aimed to assess changes in microbial biodiversity and the potential effects on plants and agricultural ecosystems. It was hypothesized that even low environmental concentrations of tetracycline could significantly alter the soil and rhizosphere microbiome. The research focused particularly on the taxonomic analysis of bacteria and the identification of pathogens, providing a better understanding of the ecological consequences of antibiotic presence in the environment. This study represents the first systematic approach to assessing microbiological dynamics in this context, offering valuable insights for plant physiology and public health.

## 2. Results and Discussion

Organic fertilizers are commonly used in agricultural production due to their low cost and high nutrient content, which improve the soil quality and crop yield. Both manure and activated sludge can contain antibiotic residues that affect the soil microbiome composition and induce antibiotic resistance in the soil [31]. To simulate real environmental conditions, soil collected from a field regularly fertilized with chicken manure was used as the control sample in this study. Although the soil was confirmed to be free of antibiotics, previous work by our team detected tetracycline in nearby groundwater [32]. Pharmaceuticals undergo various transformations in soil, and the degree of drug adsorption to soil particles depends on the chemical structure, the polarity of the drug, and the soil’s physicochemical properties. The soil used in this study had a low organic carbon content (<1.2 g/kg), resulting in a reduced ability to retain contaminants and an increased risk of leaching into deeper soil layers and/or groundwater [32]. The presence of tetracycline in the groundwater indicates its use in poultry farming, suggesting that the microbiome of soil fertilized with manure from these farms would be altered. A total of 92,009, 68,855, and 40,611 paired reads (pairs joined) were obtained for soil samples S1, S2, and S3, respectively. A phylogenetic summary of the results is presented in Table 1.

*Alphaproteobacteria* and *Gammaproteobacteria* constituted 30.32% and 12.85% of all the identified classes, respectively. Within *Alphaproteobacteria*, three families were identified: *Micropepsaceae* (7.46%) and *Rhizobiaceae* (4.56%), responsible for nitrogen fixation and cycling in nature, and *Devosiaceae* (3.97%), which have the ability to remediate contaminated soils [33]. Within *Gammaproteobacteria*, the order *Xanthomonadales* (3.39%) was identified. The *Xanthomonadales* group includes both plant and human pathogens, as well as non-pathogenic environmental bacteria [34]. Species within the genus *Stenotrophomonas* from the *Xanthomonadales* group are multidrug-resistant opportunistic pathogens that are responsible for nosocomial infections in patients with immunodeficiencies. *Stenotrophomonas maltophilia* is among the top ten pathogens causing nosocomial infections. The main cases of this bacterium being isolated are: bacteremia, wound infection, meningitis, urinary tract infections, and pneumonia [35,36]. In the study, we did not detect bacteria from the *Xanthomonadales* group at the species level; however, it is worth monitoring the bacteria from this group.

**Figure 1:**
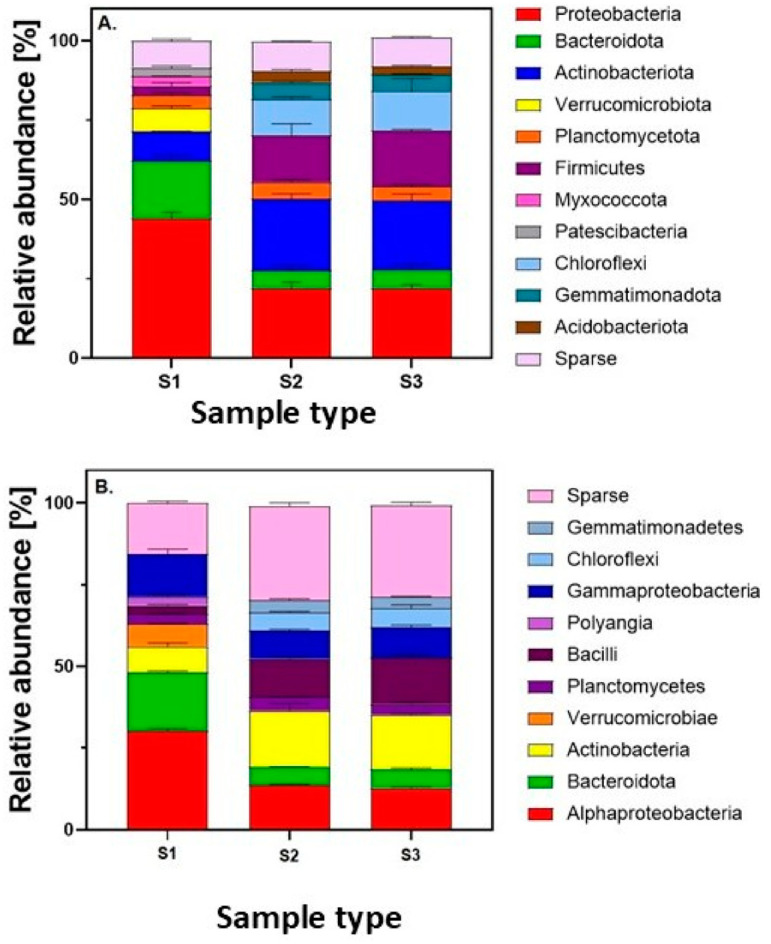
Relative abundance (%) of the soil bacterial community at the phylum (**A**) and class (**B**) levels. S1—soil without tetracycline addition; S2—soil with 5 mg/kg tetracycline addition; S3—soil with 25 mg/kg tetracycline addition. Taxa that comprised less than 5% of the total classified reads were grouped and labeled as “Sparse”.

The second most abundant bacterial group at the phylum level was *Bacteroidota* (Figure 1A). Within this phylum, the class *Bacteroidia* (17.91%) and three families were identified: *Flavobacteriaceae* (6.68%), *Chitinophagaceae* (4.59%), and *Sphingobacteriaceae* (3.13%). The presence of these bacteria has been previously reported in agricultural soils in Poland [37]. Special attention is given to the occurrence of the *Flavobacteriaceae* family and the genus *Flavobacterium*. These bacteria participate in the degradation of complex organic compounds, such as pesticides and insecticides [38], and are considered indicators of good soil quality [37]. These bacteria are also important components of the rhizosphere and phyllosphere microbiomes [39]. On the other hand, a positive correlation between *Flavobacteriaceae* and the transmission of antibiotic resistance genes in soils, including those for tetracycline resistance, has been demonstrated [40]. *Flavobacterium* bacteria can also cause infections in humans and animals [38]. *Flavobacterium* species can cause disease in fish; human infections are extremely rare. Only a few cases of opportunistic infections have been described in humans, including pneumonia, septicemia, and spontaneous bacterial peritonitis caused by the species: *Flavobacterium ceti* and *Flavobacterium lindanitolerans* [41,42,43,44].

The third most dominant bacterial group at the phylum level was *Actinobacteriota* (9.35%). Among them, the class *Actinobacteria* (7.86%) was identified. However, the abundance of this bacterial group in the soil was lower than the values reported in agricultural soils [38]. Results obtained by Minkina et al. [45] indicate that the fertilization of soils with chicken manure increases the abundance of bacteria from the *Proteobacteria* and *Bacteroidetes* groups while decreasing the abundance of *Actinobacteria*, which aligns with our findings. *Proteobacteria* and *Bacteroidetes*, along with *Firmicutes*, are components of the chicken microbiome [46]. *Firmicutes* are among the most commonly found bacterial groups in animal manure. However, our results show that in soil fertilized with manure, *Firmicutes* accounted for only 3.06% of all identified phyla. The lower abundance of *Firmicutes* in soils may be due to their reduced ability to utilize carbohydrates from manure compared with *Proteobacteria* [47]. Laconi et al. [48] suggest that the increase in *Firmicutes* abundance in manure-fertilized soils may only be a temporary effect. Additionally, the abundance of *Firmicutes* in soils depends on the soil’s physicochemical properties and agricultural practices [49].

Bacteria from the phyla *Verrucomicrobiota*, *Planctomycetota*, *Myxococcota*, and *Patescibacteria* accounted for 7.34%, 4.03%, 2.98%, and 2.72% of all identified phyla, respectively. The taxonomic profile of the soil at the phylum and class levels is presented in Figure 1. The taxonomic profile of the soil at the order and family levels is presented in Table 2. Detailed statistical data are provided in Tables S1–S9 in the Supplementary Materials. Additionally, Table S9 in the Supplementary Materials presents the calculations of the Chi-square (Χ^2^) test for the bacterial orders and families colonizing the lettuce rhizosphere. The obtained data provided a strong statistical confirmation of the relationship between the composition of bacterial communities inhabiting the lettuce rhizosphere and the applied dose of tetracycline. These findings highlight a significant impact of antibiotic exposure on microbial diversity at both the order and family levels, further emphasizing the dose-dependent shifts in the microbial structure within the rhizosphere.

The addition of tetracycline caused significant changes in the soil microbiome composition, and this effect was not dependent on the concentration of the antibiotic (Figure 1). Both lower (5 mg/kg) and higher (25 mg/kg) concentrations of the antibiotic resulted in similar soil microbiome compositions, which significantly differed from the microbiome of soil without the addition of antibiotics. The most dominant bacterial group at the phylum level was *Actinobacteriota* (22%), whose abundance in tetracycline-treated soils increased significantly compared with soil without the antibiotic (Figure 1A). The presence of *Actinobacteriota* in the soil is ecologically important as these bacteria are involved in the metabolism of organic matter and exhibit remediation capabilities [50]. On the other hand, many members of this phylum are significant human pathogens [51]. Within the *Actinobacteria* class, two orders were identified—*Micrococcales* (6.66%) and *Propionibacteriales* (3.88%)—as well as two families—*Micrococcaceae* (2.57%) and *Nocardoidiaceae* (3.86%). *Micrococcaceae* bacteria have the ability to degrade aromatic compounds in soils [52], which explains their increased abundance in tetracycline-treated soil. Fan et al. [53] also indicate that *Micrococcaceae* are carriers of tetracycline resistance genes in the soil–rhizosphere–plant internal tissue system, and this family is particularly rich in mobile genetic elements. This study examines the taxonomic profile of bacteria following exposure to tetracycline, while antibiotic resistance genes will be determined in a subsequent study. *Nocardoidiaceae* bacteria also have remediation capabilities and are detected in contaminated environments [54]. Studies show that these bacteria can degrade various xenobiotics, including ibuprofen [55], sulfamethoxazole [56], and carbamazepine [57]. An increase in the abundance of antibiotic-degrading bacteria in soils may be a factor in the spread of antibiotic resistance [58]. *Nocardia* spp. bacteria can also cause infections in humans, such as nocardiosis, including skin and pulmonary infections, especially in immunocompromised individuals [59]. In soil treated with 5 mg/kg tetracycline, the presence of *Mycobacterium tuberculosis*, a member of the *Actinobacteria* class, was also detected (Table 2). Besides *Mycobacterium tuberculosis*, which had a read frequency of 222, the most frequently occurring species identified were *Flavobacterium pectinovorum* and *Sporosarcina ureae*, with read frequencies of 4716, 1011, and 581 for soils S1, S2, and S3, respectively. Notably, a decreasing read frequency was observed with an increasing tetracycline dose.

*Mycobacterium tuberculosis* is a dangerous human pathogen causing tuberculosis. In 2022, 10.6 million people worldwide contracted tuberculosis, and 1.3 million patients died [60]. *Tuberculosis* bacteria are primarily transmitted through aerosol, in direct contact with an infected person. However, Velayati et al. [61] indicate that tuberculosis bacteria can also be transmitted without contact with an infected person. Small droplets of sputum (1–3 μm) coughed out by the patient after losing water turn into droplet nuclei and then into bacterial dust, which stays in the air for a long time in the form of an aerosol. Due to the typical structure of mycobacteria rich in lipids in the cell wall, the strong tolerance of tuberculosis bacteria benefits the survival for 10 months in dry sputum and 5 months in water [62]. *M. tuberculosis* has previously been isolated from sewage sludge, wastewater, and soils [63], with patient excrement being indicated as a source of pathogens. To date, few studies have assessed the transmission of *M. tuberculosis* from the environment to humans, and the sources of these pathogens in soil are not fully understood. The detection of this pathogen in tetracycline-treated soil raises particular concerns. *M. tuberculosis* is known to exhibit antibiotic resistance [64], and multidrug-resistant tuberculosis is one of the most serious public health threats [60]. Although this pathogen was not detected in soil treated with the highest concentration of tetracycline, *Mycobacterium holsaticum* was identified. The genus *Mycobacterium* includes over 140 species, and many new species have been described from the environment in recent years [65]. *Mycobacterium holsaticum* is rarely reported as a cause of human infections. This strain was first isolated from clinical cases in Germany [66]. There are also case reports identifying this microorganism as a causative agent of infections. Verghese et al. [67] described a case of intestinal infection caused by *M. holsaticum*. De Lima et al. [68] isolated *M. holsaticum* from the respiratory tract of patients with pulmonary tuberculosis, highlighting the need for the identification of potential infections caused by bacteria not belonging to the *Mycobacterium tuberculosis* complex (non-tuberculosis bacteria). The transmission of *Mycobacterium tuberculosis* from soil to humans is possible but rare. These bacteria can survive in soil due to their lipid-rich cell walls, which protect them from desiccation and environmental stress. Soil contaminated with sputum or waste from infected individuals can pose an exposure risk, particularly under specific conditions, such as inhaling dust or contact with wounds. *M. tuberculosis* has been detected in soil samples from areas inhabited by animals, suggesting potential links between infected environments, animals, and humans. Examples from Africa show genetic similarities between *M. tuberculosis* strains in soil and those found in human populations. Nevertheless, the primary route of *M. tuberculosis* transmission remains the inhalation of droplets from infected individuals. Advances in detection methods, such as genome sequencing, could enhance the understanding and assessment of environmental risks posed by *M. tuberculosis* in soil [69]. In soil treated with the lowest concentration of tetracycline, *Actinomadura geliboluensis* was also detected. Although its pathogenicity in humans has not been reported to date, it was isolated from a patient with pneumonia in 2023 [70]. The occurrence frequency of *Stenotrophomonas maltophilia*, an opportunistic pathogenic bacterium, was 73, whereas the occurrence frequency of *Mycobacterium holsaticum* was 68 (Table 2). The most frequently identified genera are microorganisms that play a key role in environmental and biological processes. They contribute to nutrient cycling (*Bradyrhizobium japonicum*, *Sporosarcina ureae*), biodegradation (*Flavobacterium pectinovorum*, *Arthrobacter crystallopoietes*), and bioremediation (*Flavobacterium arsenitoxidans*, *Rhodanobacter spathiphylli*).

The accumulation of antibiotics present in consumed vegetables poses a threat and potential health risk to humans through the food chain [71,72]. Antibiotic concentrations in vegetables are relatively low, generally ranging from trace amounts to several dozen micrograms per kilogram [73,74]. These concentrations fall within the subinhibitory range, which is significantly lower than the minimum inhibitory concentration (MIC). However, numerous studies indicate that even exposure to such low concentrations promotes the selection of antibiotic-resistant bacterial strains [75,76,77]. This means that in the presence of antibiotics, only mutated bacterial cells that have acquired resistance survive, while susceptible bacteria perish. Consequently, resistant strains become dominant within the microbiome, whereas susceptible populations decline. As demonstrated by Pomati et al. [78] and Subirats et al. [79] in clinical and veterinary studies, even low antibiotic concentrations in food can contribute to the selection of resistant bacteria. However, it should be unequivocally stated that studies regarding their impact on the human gut microbiome remain limited. In studies in patients exposed to antibiotics, Duan et al. [80] demonstrated a positive correlation between antibiotic exposure and the presence of antibiotic resistance genes (ARGs), along with a negative correlation between antibiotic exposure and bacterial diversity in the gut microbiome.

In tetracycline-treated soil, a 21% decrease in the abundance of *Proteobacteria* was observed (Figure 1A). Xiong et al. [81] also reported a reduction in the abundance of *Proteobacteria* in soils treated with tetracycline. The authors suggest that the decrease in *Proteobacteria* abundance in response to tetracycline treatment indicates the occurrence of antibiotic resistance. In our case, however, this aspect requires further investigation. Among *Proteobacteria*, a decrease in the abundance of the *Rhizobiales* and *Burkholderiales* orders was observed. The reduction in these orders may have significant consequences for soil health and plant growth, as these bacteria play key roles in soil ecosystems. Tetracycline-treated soils showed a significant increase in the abundance of *Firmicutes* bacteria (with an increase of 11.6% and 14.7% in soils with 5 and 25 mg/kg tetracycline, respectively). Blau et al. [82] demonstrated an increase in this taxon in soils fertilized with manure supplemented with doxycycline, indicating that *Firmicutes* are responsible for the spread of ARGs in the environment. Within the *Firmicutes* phylum, one class—*Bacilli*—and one order—*Bacillales*—were identified (Figure 1B, Table 3). *Bacillales* bacteria perform many important functions in soils, including participation in nutrient cycling and exhibiting remediation properties [83]. On the other hand, an increase in the abundance of these taxa following tetracycline application may indicate the selection of antibiotic-resistant strains. Within the *Bacillales* order, two families—*Planococcaceae* and *Bacillaceae*—were identified (Table 3). The presence of *Chloroflexi* bacteria was also observed in tetracycline-treated soils (Figure 1), which were not identified in soil without the antibiotic addition. Although the exact function of these bacteria in soil is unknown, they survive in low-fertility soils [84]. In soils treated with antibiotics, a decrease in the abundance of *Bacteroidota* (by 12% compared with soil without tetracycline) was also observed. These bacteria are an important group of soil microorganisms, and a reduction in their abundance may have negative consequences for both the soil and the plants grown in it. In tetracycline-treated soils, *Gemmatimonadota* bacteria were also identified (Figure 1A). These bacteria are widely distributed in the environment. Their cosmopolitan distribution in various soils suggests that they utilize a broad range of nutrients [85]. Their presence in tetracycline-treated soils was also demonstrated by Zheng et al. [86]. The increased abundance of these bacteria in tetracycline-treated soils may be related to their use of tetracycline as a carbon source. Li et al. [87] demonstrated that *Gemmatimonadota* bacteria were carriers of antibiotic resistance genes in soil treated with sulfamethoxazole. The relationship between the increased abundance of these bacteria in tetracycline-treated soils and their potential use of tetracycline as a carbon source will be further investigated. In addition, the role of *Gemmatimonadota* bacteria as carriers of antibiotic resistance genes will be addressed in future studies.

Changes in the composition of soil microorganism populations can also lead to changes in the rhizosphere microbiome. The rhizosphere is an environment that is particularly rich in microorganisms. Rhizosphere bacteria play a crucial role in maintaining plant health [88] by improving the availability of nutrients, including nitrogen [89] and phosphorus [90]. Additionally, rhizosphere bacteria can produce plant hormones (auxins, cytokinins, and gibberellins) that stimulate plant growth and development [33].

Furthermore, they protect plants from pathogens by producing antibiotic substances and inducing systemic resistance. Increasingly, research highlights the role of fresh vegetables and fruits in shaping the human microbiome [91]. Among these, lettuce is one of the most commonly consumed raw vegetables [92], with a global cultivation area of 1.24 million hectares [93].

Therefore, it is important to study the microbiome of frequently consumed plants, especially grown in environments contaminated with antibiotics. A total of 56,862, 69,185, and 44,995 paired reads (pairs joined) were obtained for the rhizosphere microbiome of lettuce grown in soil without antibiotic addition (R1), with 5 mg/kg tetracycline addition (R2), and with 25 mg/kg tetracycline addition (R3), respectively. A phylogenetic summary of the results is presented in Table 4.

**Figure 2:**
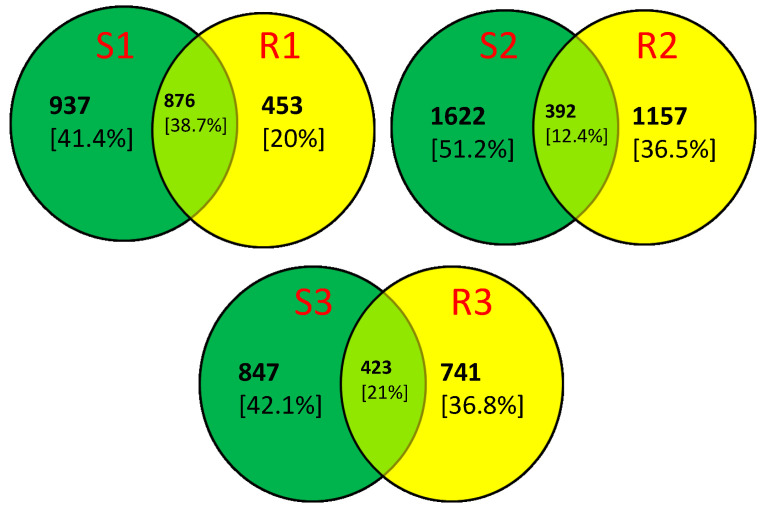
Shared ASVs between soil and the lettuce rhizosphere. S1—soil without tetracycline addition, S2—soil with 5 mg/kg tetracycline addition, S3—soil with 25 mg/kg tetracycline addition, R1—rhizosphere of lettuce grown on soil without tetracycline addition, R2—rhizosphere of lettuce grown on soil with 5 mg/kg tetracycline addition, R3—rhizosphere of lettuce grown on soil with 25 mg/kg tetracycline addition.

Although tetracycline significantly alters the composition of microorganisms inhabiting contaminated soils, this effect is not as pronounced in the rhizosphere. The impact of antibiotics on rhizosphere microorganisms is influenced by factors such as the interaction between antibiotic molecules and root exudates [94], as well as the physical and chemical properties of the soil [95]. Besides soil microorganism composition, other factors shaping the plant rhizosphere include the soil type, pH [96], and plant developmental stage [4]. Žiarovská et al. [97] demonstrated that the composition of microorganisms varies depending on the lettuce species. Additionally, the seed microbiome may influence the final composition of the rhizosphere microorganism population [98]. Based on ASV data, it was found that only 12.4% and 21% of ASVs in the rhizosphere microbiome of lettuce treated with tetracycline at concentrations of 5 mg/kg and 25 mg/kg, respectively, originated from the soil (Figure 2). In control samples (S1, R1), 38.7% of ASVs were common between the soil and rhizosphere (Figure 2). Under stress conditions, plants alter the chemical composition of root exudates, attracting microorganisms that support plant health [99]. Huang et al. [100] found that rhizosphere bacteria under antibiotic-induced stress enhance their community structure by dispersing antibiotics in the soil and favoring the growth of key species that support plant growth. Therefore, the final composition of the rhizosphere microbiome depends on specific interactions between antibiotics, the soil, and the plants.

**Figure 3:**
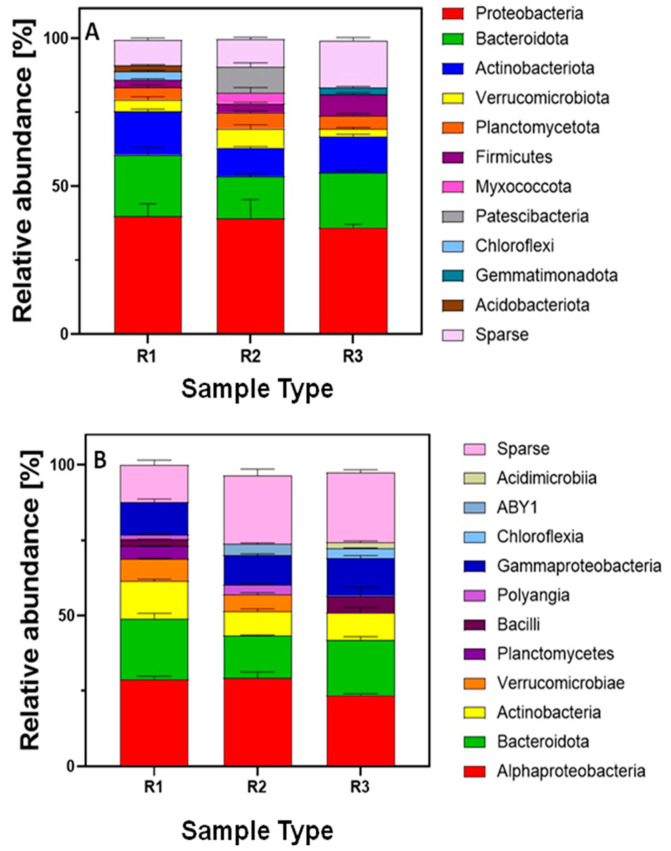
Relative abundance (%) of the lettuce rhizosphere bacterial community at the phylum (**A**) and class (**B**) levels. R1—lettuce grown in soil without tetracycline addition, R2—lettuce grown in soil with 5 mg/kg tetracycline addition, R3—lettuce grown in soil with 25 mg/kg tetracycline addition. Taxa that made up less than 5% of the total classified reads were grouped and labeled as “Sparse”.

In the lettuce rhizosphere, bacteria from 11 phyla were identified: *Proteobacteria*, *Bacteroidota*, *Actinobacteriota*, *Verrucomicrobiota*, *Planctomycetota*, *Firmicutes*, *Myxococcota*, *Patescibacteria*, *Chloroflexi*, *Gemmatimonadota*, and *Acidobacteriota*, in varying proportions (Figure 3). Pandiyan et al. [101] indicate that at the phylum level, plant microbiomes show significant similarities. However, lower taxonomic levels are shaped by environmental conditions and differ from each other. In the lettuce rhizosphere, the relative abundance of *Proteobacteria* was higher compared with the soil (Figure 1, Figure 2). Similar results were also obtained by Schreiter et al. [96]. The plant rhizosphere is preferentially colonized by *Proteobacteria*, *Bacteroidetes*, and *Actinobacteriota* because these bacteria actively utilize root exudates [102]. These taxa also dominate within the roots [3]. Our results indicate *Proteobacteria*, *Bacteroidetes*, and *Actinobacteria* as dominant bacterial phyla in the lettuce rhizosphere (Figure 2), including those treated with tetracycline. At the phylum level, the most dominant bacterial group in each sample was *Proteobacteria* (Figure 3), and the addition of tetracycline did not significantly alter the structure of this bacterial group. Within Proteobacteria, two classes were identified: *Alphaproteobacteria* and *Gammaproteobacteria* (Figure 3); five orders: *Rhizobiales*, *Micropepsales*, *Caulobacterales*, *Burkholderiales*, and *Xanthomonadales*; and five families: *Rhizobiaceae*, *Devosiaceae*, *Xanthobacteraceae*, *Comamonadaceae*, and *Rhodanobacteraceae* (Table 5). These bacteria perform diverse and complex functions in the soil ecosystem, positively influencing plant health and growth. Plant growth-promoting bacteria (PGPB) play a key role in maintaining plant health, especially in contaminated environments. However, there is growing concern about the presence of antibiotic resistance genes among these bacteria, particularly within the *Rhizobiales* and *Burkholderiales* taxa [103]. These taxa should therefore be monitored in plants grown in environments contaminated with antibiotics. These taxa constituted 13.43% and 5.30% of the identified orders in the rhizosphere of R1 and R2, respectively. Among *Burkholderiales*, an increase in abundance was observed in the rhizosphere with rising tetracycline concentrations in the soil. According to Wicaksono et al. [5], *Burkholderiales* bacteria associated with vegetables and fruits are a functional component of the human microbiome. This taxon includes both health-promoting species and pathogens, as well as antibiotic-resistant strains. Among them, *Burkholderia cepacia* is a plant phytogen and is known as a hardy and versatile organism. Over the past two decades it has emerged as a pathogen in the cystic fibrosis (CF) community, with devastating effects. Pulmonary colonization can lead to an accelerated decline in lung function. *B. cepacia* is inherently resistant to multiple antibiotics and highly transmissible and virulent strains have been identified [104]. Therefore, the presence of these bacteria in plants growing in contaminated environments should be closely monitored. The *Bacteroidota* phylum in the rhizosphere of lettuce grown without antibiotic addition was represented by the class *Bacteroidia* (Figure 3), the order *Flavobacteriales*, the family *Flavobacteriaceae* (Table 5), and the genus *Flavobacterium*. Žiarovská et al. [97] also identified the genus *Flavobacterium* as one of the most abundant in the lettuce rhizosphere. These bacteria likely play a role in maintaining plant health and are considered growth-promoting taxa [39]. They are also widely distributed in the plant phyllosphere. Our results suggest that these bacteria survive in the rhizosphere of plants growing in tetracycline-contaminated environments, despite a decrease in their abundance in contaminated soils.

Although these taxa have a positive impact on plants, their ability to survive in antibiotic-contaminated environments raises concerns about the spread of antibiotic resistance. These bacteria are considered carriers of tet (A) resistance genes among bacterial communities [105]. In the rhizosphere of plants R2 and R3, an increase in the abundance of the *Chitinophagales* taxon was also observed. The increase in chitin-degrading taxa in the rhizosphere of lettuce treated with antibiotics may be related to the bacteriostatic effect of tetracycline and the associated fungal overgrowth [82]. The *Actinobacteriota* phylum was represented by bacteria from the orders *Micrococcales* and *Propionibacteriales* and the family *Nocardioidaceae* (Table 5). The presence of these bacteria has been previously reported in soil treated with tetracycline. However, in the rhizosphere of plants treated with tetracycline, the presence of *Mycobacterium tuberculosis*, which was present in the soil, was not observed. The most significant changes in the rhizosphere due to tetracycline were observed in the *Firmicutes* bacteria (Figure 3). The abundance of this phylum increased in the rhizosphere of lettuce grown in tetracycline-contaminated soil, and this increase was proportional to the rising concentration of the antibiotic. *Firmicutes* are widely distributed in the lettuce microbiome [106], and the phyllosphere (aboveground plant parts) is typically represented by microorganisms that thrive in the given environment [101]. Zhang et al. [107] demonstrated a positive correlation between *Firmicutes* and the presence of the tetX gene in the rhizosphere. The increase in the abundance of this taxon in the plant rhizosphere may be related to the occurrence of antibiotic resistance. However, this aspect requires further investigation. The *Gemmatimonadota* phylum has also been associated with the presence of tetracycline resistance genes (tetT) [108]. An increase in the abundance of this taxon was observed in the rhizosphere of lettuce treated with the highest concentration of tetracycline (Figure 3). Increasing evidence suggests that minimally processed, raw lettuce can be a pathway for the introduction of pathogens and ARGs into the human body [109]. However, our results do not indicate the presence of pathogens in the lettuce rhizosphere.

It should be noted that only a few percent of the strains in the lettuce rhizosphere were identified in this study. However, the detection of *M. tuberculosis* in tetracycline-treated soil is concerning. The colonization of plants by human pathogens is strongly dependent on the composition and diversity of the plant microbiome. Lim et al. [110] demonstrated that pathogens such as *Salmonella* are capable of overcoming plant immune defenses. Whether this ability is present in *Mycobacterium tuberculosis* remains unknown, but cultivating plants in environments contaminated with antibiotics raises concerns regarding their microbiological quality. On the other hand, Yu et al. [111] identified a high prevalence (60%) of ARGs among bacteria that are considered beneficial to plants, whose role involves stimulating and supporting plant growth. In our study, beneficial organisms for plants also dominate, although their proportion changes (Table 2 and Table 6). The possibility of this pathogen entering plant tissues should be investigated. The detailed composition of microorganisms inhabiting the rhizosphere of lettuce grown in tetracycline-contaminated environments at the phylum and class levels is shown in Figure 3. The taxonomic profile of the rhizosphere of lettuce treated with tetracycline at the order and family levels is presented in Table 5.

Table 6 presents the frequency of microbial genus reads along with their corresponding order and family, fully confirming the observed reduction. In the rhizosphere, the dominant microorganism was *Flavobacterium pectinovorum*, with read frequencies of 4662, 3625, and 1360 for lettuce grown without tetracycline and with doses of 5 mg/kg and 25 mg/kg, respectively. Microorganisms present in the rhizosphere play important roles in the environment, particularly in the nitrogen cycle, biodegradation, and bioremediation, and they are not pathogenic to humans.

## 3. Materials and Methods

### 3.1. Lettuce Growing Conditions

The soil was collected in early spring 2021 from an agricultural field before the application of a fresh batch of manure. The field had been regularly fertilized with chicken manure in previous growing seasons. The agricultural field is located in the Warmian–Masurian Voivodeship. After collection, the samples were transported to the laboratory and stored at 4 °C before analysis. The soil was confirmed to be free of antibiotics. A detailed characterization of the soil is presented in the work by Krupka et al. [32]. Seeds of lettuce (*Lactuca sativa* L. var. Takoda) were sown in seed trays measuring 24 × 24 mm. The plants were then grown for 4 weeks in climate chambers (POL-EKO, Wodzisław Śląski, Poland) at a temperature of 20 °C/15 °C day/night, with a 16/8 h photoperiod, and a light intensity of 8000 lx. Subsequently, 2 kg of soil was added to pots with a diameter of 17 cm, and lettuce seedlings (one seedling per pot) were transplanted. The plants were then watered with 15 mL of an aqueous solution of tetracycline–HCl (Sigma-Aldrich, Burlington, MA, USA) at concentrations of 5 mg/kg (S2) and 25 mg/kg (S3). According to Ji et al. [112], tetracycline is detected in concentrations ranging from 5 to 25 mg/kg in areas with increased agricultural activity. The control group consisted of lettuce grown in soil without tetracycline (S1), watered with distilled water. A total of five replicates were obtained for each treatment. The plants were grown for an additional 4 weeks under the same conditions before further analyses were conducted.

### 3.2. DNA Isolation from Soil and Rhizosphere

Plants were removed from the pots, and the leaves and roots were separated using sterile scissors. To minimize local variability in bacterial populations, 10 lettuce plants were cultivated for each treatment. Soil tightly adhering to the roots was considered the rhizosphere. Rhizosphere samples were collected from each plant and pooled to create a single, homogeneous composite sample representative of the rhizosphere. A similar approach was applied to soil samples to obtain a representative soil sample. DNA was isolated from 250 mg of soil and rhizosphere using the DNeasy Power Pro Soil Kit (Qiagen INC, Montgomery County, MD, USA), following the manufacturer’s protocol. The quantity of isolated DNA was measured using a NanoDrop 1000 spectrophotometer (NanoDrop Technologies, Wilmington, NC, USA). The purity of the isolated DNA was checked using the OD 260/280 and OD 260/230 parameters (NanoDrop Technologies, Wilmington, NC, USA). The resulting genetic material was suspended in Tris–HCl buffer at pH 7 and stored at −20 °C until further analysis. DNA isolation was performed in five replicates.

### 3.3. Metagenomic Analysis of Soil and Rhizosphere Samples

The metagenomic analysis of the samples was conducted by an external company (Genomed S.A., Warsaw, Poland). The bacterial population analysis was based on the hypervariable V3–V4 region of the 16S rRNA gene. Specific primer sequences 341F and 785R were used for the amplification of the selected region and library preparation. The PCR reaction was performed using the Q5 Hot Start High-Fidelity 2X Master Mix (New England Biolabs Inc., Ipswich, MA, USA), according to the manufacturer’s recommendations. Sequencing was performed on a MiSeq system using paired-end (PE) technology, 2 × 300 nt, with an Illumina v3 kit. The bioinformatic analysis, which ensured the classification of reads to the species level, was conducted using the QIIME 2 software package based on the Silva 138 reference sequence database. The DADA2 package (Version 1.3) was also used to differentiate between biologically derived sequences and those newly generated during sequencing. This package was also utilized to extract unique biologically derived sequences, known as ASVs (amplicon sequence variants). For the taxonomic analysis, three independent replicates were prepared. A one-way analysis of variance (ANOVA) was conducted to evaluate significant differences between bacterial communities in the rhizosphere and the soil. Post-hoc tests were also applied. Additionally, calculations of the Chi-square (Χ^2^) test for the bacterial orders and families, including Yates’ correction, were performed. The predicted values were based on data from the no-antibiotic condition, while the variables were the data for 5 and 25 mg/kg tetracycline. The calculations were carried out using the Preacher online program [113] (http://quantpsy.org, accessed on 3 March 2025).

## 4. Conclusions

1. Tetracycline at concentrations of 5 and 25 mg/kg significantly alters the soil microbiome at various taxonomic levels, and this effect is noticeable even after applying the lowest concentration of the antibiotic.
2. The presence of *M. tuberculosis* in soil treated with 5 mg/kg tetracycline may suggest that tetracycline affects the microbiome in a way that favors the survival of this pathogen in the environment. This finding requires further investigation to understand the mechanisms allowing *M. tuberculosis* to persist in soil environments, the potential transfer to plant tissues, and public health risks.
3. The addition of tetracycline did not induce significant changes in the taxonomic profile of the rhizosphere microorganisms. This suggests that plants may influence the stabilization of the rhizosphere microbiome.
4. The increased abundance of *Firmicutes* bacteria after tetracycline treatment in soil and rhizosphere suggests that this taxon should be particularly monitored in plants growing in antibiotic-contaminated environments.

The use of tetracycline and other antibiotics in agriculture can lead to lasting changes in the soil microbiome, which may have long-term consequences for soil ecosystem health and crop plants. Given the observed changes, further research is necessary to better understand the mechanisms underlying microorganism adaptation to tetracycline and their long-term effects on soil ecosystems and plant health. This study serves as a preliminary investigation for more advanced research, including the detection of antibiotic resistance genes in plant tissues, which will significantly contribute to protecting consumers who eat fresh, unprocessed lettuce leaves.

## Figures and Tables

**Table 1:** Percentage of reads assigned to appropriate taxonomic levels for analyzed samples. S1—soil without tetracycline addition; S2—soil with 5 mg/kg tetracycline addition; S3—soil with 25 mg/kg tetracycline addition.

Sample	Kingdom	Phylum	Class	Order	Family	Genus	Species
S1	100	99.99	99.96	99.47	96.15	76.45	9.607
S2	100	99.93	99.86	98.78	94.24	80.43	6.89
S3	100	99.98	99.86	98.88	95.08	82.86	6.78

**Table 2:** Read frequency (number of reads) of the microbial genus and their taxonomic classification at the family and order levels depending on the applied tetracycline dose. S1—soil without tetracycline addition; S2—soil with 5 mg/kg tetracycline addition; S3—soil with 25 mg/kg tetracycline addition.

Order	Family	Genus	Number of Reads
S1			
*Bacteroidota*	*Flavobacterium*	*Flavobacterium pectinovorum*	4716
*Proteobacteria*	*Bradyrhizobium*	*Bradyrhizobium japonicum*	324
*Actinobacteriota*	*Arthrobacter*	*Arthrobacter crystallopoietes*	193
*Proteobacteria*	*Rhodanobacter*	*Rhodanobacter spathiphylli*	165
*Firmicutes*	*Sporosarcina*	*Sporosarcina ureae*	144
*Cyanobacteria*	*Chloroplast*	*Lactuca sativa*	121
*Proteobacteria*	*Luteimonas*	*Lysobacter pocheonensis*	113
*Bacteroidota*	*Flavobacterium*	*Flavobacterium arsenitoxidans*	108
S2			
*Firmicutes*	*Sporosarcina*	*Sporosarcina ureae*	1011
*Actinobacteriota*	*Arthrobacter*	*Arthrobacter crystallopoietes*	381
*Actinobacteriota*	*Actinomadura*	*Mycobacterium tuberculosis*	222
*Proteobacteria*	*Bradyrhizobium*	*Bradyrhizobium japonicum*	203
*Firmicutes*	*Sporosarcina*	*Sporosarcina psychrophila*	147
*Firmicutes*	*Oceanobacillus*	*Oceanobacillus indicireducens*	137
*Myxococcota*	*Polyangiaceae*	*Sorangium cellulosum*	129
*Actinobacteriota*	*Actinomadura*	*Actinomadura geliboluensis*	118
S3			
*Firmicutes*	*Sporosarcina*	*Sporosarcina ureae*	581
*Actinobacteriota*	*Arthrobacter*	*Arthrobacter crystallopoietes*	216
*Firmicutes*	*Sporosarcina*	*Sporosarcina psychrophila*	139
*Firmicutes*	*Oceanobacillus*	*Oceanobacillus indicireducens*	104
*Proteobacteria*	*Bradyrhizobium*	*Bradyrhizobium japonicum*	90
*Actinobacteriota*	*Agromyces*	*Agromyces neolithicus*	80
*Proteobacteria*	*Stenotrophomonas*	*Stenotrophomonas maltophilia*	73
*Actinobacteriota*	*Mycobacterium*	*Mycobacterium holsaticum*	68

**Table 3:** Relative abundance (%) of soil bacterial community at the order and family levels. S1—soil without tetracycline addition, S2—soil with 5 mg/kg tetracycline addition, S3—soil with 25 mg/kg tetracycline addition. Grouping results G1, G2, G3 for S1, S2, S3. Results of the ANOVA with Tukey’s test (α = 0.05) for multiple comparisons, conducted using GraphPad Prism 10.4.0.

**Order**	**S1**	**S2**	**S3**	**SG1**	**GG2**	**CG3**
*Rhizobiales*	13.16 ± 0.97	7.51 ± 0.57	6.70 ± 0.40	A	A	B
*Micropepsales*	7.46 ± 0.56	<1 ± 0.09	6.54 ± 0.39	A	B	A
*Burkholderiales*	7.29 ± 0.89	3.68 ± 0.41	3.94 ± 0.54	A	A	A
*Flavobacteriales*	6.8 ± 0.85	<1 ± 0.08	<1 ± 0.08	A	B	B
*Chitinophagales*	5.34 ± 0.4	<1 ± 0.09	<1 ± 0.10	A	B	B
*Opitutales*	3.87 ± 0.37	<1 ± 0.09	<1 ± 0.08	A	B	B
*Sphingobacteriales*	3.42 ± 1.18	<1 ± 0.03	<1 ± 0.06	A	B	B
*Xanthomodales*	3.39 ± 0.78	<1 ± 0.03	<1 ± 0.04	A	B	B
*Bacillales*	<1 ± 0.06	10.25 ± 0.33	12.58 ± 0.37	B	A	A
*Micrococcales*	<1 ± 0.06	6.66 ± 0.43	6.54 ± 0.36	B	A	A
*Thermomicrobiales*	<1 ± 0.11	5.06 ± 0.99	5.36 ± 0.37	B	A	A
*Gemmatimonadales*	<1 ± 0.07	3.78 ± 0.19	3.5 ± 0.06	B	A	A
*Gitt-GS*	<1 ± 0.05	2.87 ± 1.1	<1 ± 0.06	B	B	A
*Propionibacteriales*	<1 ± 0.05	<1 ± 0.44	3.92 ± 0.25	B	A	B
**Family**	**S1**	**S2**	**S3**	**SG1**	**GG2**	**CG3**
*Rhizobiaceae*	4.56 ± 0.48	2.79 ± 0.39	<1 ± 0.10	A	B	C
*Micropepsaceae*	7.46 ± 0.56	<1 ± 0.10	<1 ± 0.05	A	B	B
*Flavobacteriaceae*	6.68 ± 1.59	<1 ± 0.04	<1 ± 0.04	A	B	B
*Chitiniphagaceae*	4.59 ± 0.09	<1 ± 0.07	<1 ± 0.08	A	B	B
*Devosiaceae*	3.97 ± 0.82	<1 ± 0.06	<1 ± 0,04	A	B	B
*Comamonadaceae*	3.84 ± 0.31	<1 ± 0.06	<1 ± 0.04	A	B	B
*Opitutaceae*	3.81 ± 0.18	<1 ± 0.06	<1 ± 0.05	A	B	B
*Sphinhobacteriaceae*	3.13 ± 0.12	<1 ± 0.05	<1 ± 0.03	A	B	B
*Planococcaceae*	<1 ± 0.1	5.93 ± 0.89	7.31 ± 0.88	B	A	A
*Bacillaceae*	<1 ± 0.07	4.3 ± 0.26	5.26 ± 0.27	B	A	A
*Nocardioidaceae*	<1 ± 0.1	3.86 ± 0.48	3.91 ± 0.06	B	A	A
*Gemmatimonadaceae*	<1 ± 0.06	3.77 ± 0.71	3.51 ± 0.2	B	A	A
*Gitt-GS*	<1 ± 0.06	2.87 ± 0.60	2.99 ± 0.35	B	A	A
*Micrococcaceae*	<1 ± 0.07	2.57 ± 0.06	2.58 ± 0.24	B	A	A

**Table 4:** Percentage of reads assigned to appropriate taxonomic levels for analyzed samples. R1—rhizosphere of lettuce grown on soil without tetracycline addition, R2—rizosphere of lettuce grown on soil with 5 mg/kg tetracycline addition, R3—rizosphere of lettuce grown on soil with 25 mg/kg tetracycline addition.

Sample	Kingdom	Phylum	Class	Order	Family	Genus	Species
R1	100	100	99.97	99.34	95.87	76.67	13.6
R2	100	99.97	99.94	99.26	95.00	76.72	5.35
R3	100	99.89	99.84	99.13	95.96	79.05	13.43

**Table 5:** Relative abundance (%) of the rhizosphere bacterial community at the order and family levels. R1—rhizosphere of lettuce grown in soil without tetracycline addition, R2—rhizosphere of lettuce grown in soil with 5 mg/kg tetracycline addition, R3—rhizosphere of lettuce grown in soil with 25 mg/kg tetracycline addition. Grouping results G1, G2, G3 for S1, S2, S3. Results of the ANOVA with Tukey’s test (α = 0.05) for multiple comparisons, conducted using GraphPad Prism 10.4.0.

**Order**	**R1**	**R2**	**R3**	**G1**	**G2**	**G3**
*Rhizobiales*	14.94 ± 0.65	13.43 ± 0.74	11.69 ± 0.44	A	AB	B
*Micropepsales*	4.14 ± 0.35	4.63 ± 1.09	<1 ± 0.09	A	A	B
*Burkholderiales*	4.94 ± 0.81	5.29 ± 0.13	6.52 ± 0.62	A	AB	B
*Flavobacteriales*	10.4 ± 0.48	13.24 ± 0.45	10.46 ± 1.72	A	B	A
*Chitinophagales*	4.45 ± 1.00	4.95 ± 1.15	4.35 ± 0.85	A	A	A
*Opitutales*	<1 ± 0.07	<1 ± 0.09	<1 ± 0.10	A	B	A
*Sphingobacteriales*	<1 ± 0.07	4.38 ± 0.17	<1 ± 0.06	A	B	A
*Xanthomodales*	<1 ± 0.06	<1 ± 0.07	<1 ± 0.12	A	A	A
*Bacillales*	<1 ± 0.06	<1 ± 0.08	5.05 ± 0.39	A	A	B
*Micrococcales*	3.79 ± 0.24	<1 ± 0.08	3.37 ± 0.75	A	B	A
*Gemmatimonadales*	<1 ± 0.08	<1 ± 0.07	<1 ± 0.10	A	A	A
*Candidatus*	<1 ± 0.06	3.72 ± 1.1	<1 ± 0.08	A	B	A
*Propionibacteriales*	<1 ± 0.10	<1 ± 0.2	3.02 ± 0.41	A	A	B
*Caulobacterales*	3.45 ± 0.63	4.96 ± 1.97	3.09 ± 0.56	A	B	A
**Family**	**R1**	**R2**	**R3**	**G1**	**GG2**	**GG3**
*Rhizobiaceae*	5.13 ± 0.34	3.31 ± 0.67	3.79 ± 0.37	A	AB	B
*Micropepsaceae*	4.14 ± 0.27	4.63 ± 0.96	<1 ± 0.14	A	A	B
*Flavobacteriaceae*	10.16 ± 0.94	10.13 ± 0.13	10.18 ± 1.96	A	A	A
*Chitiniphagaceae*	4.12 ± 1.03	3.79 ± 0.21	3.71 ± 0.39	A	A	A
*Devosiaceae*	4.52 ± 0.71	4.55 ± 0.75	3.36 ± 0.43	A	A	A
*Comamonadaceae*	2.97 ± 0.12	<1 ± 0.13	3.47 ± 0.92	A	B	A
*Sphinhobacteriaceae*	<1 ± 0.05	4.07 ± 0.52	<1 ± 0.05	B	A	B
*Planococcaceae*	<1 ± 0.17	<1 ± 0.19	2.78 ± 0.36	B	B	A
*Nocardioidaceae*	3.00 ± 0.12	3.01 ± 0.05	3.18 ± 0.66	A	B	A
*Gemmatimonadaceae*	<1 ± 0.12	3.77 ± 1.21	3.51 ± 0.5	B	A	A
*Xanthobacteraceae*	3.25 ± 0.39	<1 ± 0.14	2.97 ± 0.25	A	B	A
*Comamonadaceae*	2.97 ± 0.37	<1 ± 0.10	<1 ± 0.08	A	B	B
*Caulobacteriaceae*	<1 ± 0.23	4.73 ± 1.95	2.73 ± 0.64	B	A	A

**Table 6:** Read frequency (number of reads) of the microbial genus and their taxonomic classification at the family and order levels depending on the applied tetracycline dose. R1—rhizosphere of lettuce grown on soil without tetracycline addition, R2—rhizosphere of lettuce grown soil with 5 mg/kg tetracycline addition, R3—rhizosphere of lettuce grown on soil with 25 mg/kg tetracycline addition.

Order	Family	Genus	Number of Reads
R1			
*Bacteroidota*	*Flavobacterium*	*Flavobacterium pectinovorum*	4662
*Actinobacteriota*	*Arthrobacter*	*Arthrobacter crystallopoietes*	265
*Proteobacteria*	*Bradyrhizobium*	*Bradyrhizobium japonicum*	217
*Bacteroidia*	*Flavobacterium*	*Flavobacterium akiainvivens*	152
*Proteobacteria*	*Rhodanobacter*	*Rhodanobacter spathiphylli*	139
*Actinobacteriota*	*Flexivirga*	*Flexivirga alba*	114
*Proteobacteria*	*Rhodanobacter*	*Rhodanobacter fulvus*	109
*Proteobacteria*	*Luteimonas*	*Lysobacter pocheonensis*	97
R2			
*Bacteroidota*	*Flavobacterium*	*Flavobacterium pectinovorum*	3625
*Firmicutes*	*Sporosarcina*	*Sporosarcina ureae*	293
*Proteobacteria*	*Bradyrhizobium*	*Bradyrhizobium japonicum*	139
*Actinobacteriota*	*Arthrobacter*	*Arthrobacter crystallopoietes*	128
*Proteobacteria*	*Rhodanobacter*	*Rhodanobacter spathiphylli*	122
*Actinobacteriota*	*Agromyces*	*Agromyces neolithicus*	88
*Cyanobacteria*	*Chloroplast*	*Lactuca sativa*	84
*Proteobacteria*	*Methylophilus*	*Methylomonas clara*	68
R3			
*Bacteroidota*	*Flavobacterium*	*Flavobacterium pectinovorum*	1360
*Firmicutes*	*Sporosarcina*	*Sporosarcina ureae*	168
*Proteobacteria*	*Bradyrhizobium*	*Bradyrhizobium japonicum*	158
*Cyanobacteria*	*Chloroplast*	*Lactuca sativa*	145
*Actinobacteriota*	*Flexivirga*	*Flexivirga alba*	118
*Proteobacteria*	*Beijerinckiaceae*	*Chelatococcus asaccharovorans*	99
*Planctomycetota*	*Paludisphaer*	*Paludisphaera borealis*	86
*Actinobacteriota*	*Nocardioides*	*Nocardioides tritolerans*	81

## Data Availability

The data presented in this study are available on request from the corresponding author.

## Supplementary Materials

The following supporting information can be downloaded at: https://www.mdpi.com/article/10.3390/ijms26072854/s1.
