# Supplementary material for: The Impact of Tetracycline on the Soil Microbiome and the Rhizosphere of Lettuce (Lactuca sativa L.)
Source: Int J Mol Sci. 2025 Mar 21;26(7):2854. doi: 10.3390/ijms26072854 (PMC11988489; doi:10.3390/ijms26072854)
Supplement: Supplementary file 1 [file ijms-26-02854-s001.zip › ijms-3498862-supplementary.pdf]

Supplementary Materials

Table S1. Statistical analysis of bacterial communities in the rhizosphere (for order). Results of ANOVA with Tukey's Test (α = 0.05) for multiple comparisons, conducted using GraphPad Prism 10.4.0. The experimental conditions are defined as follows: R1 – without tetracycline, R2 – 5 mg/kg tetracycline, R3 – 25mg/kg tetracycline. The table also includes grouping results (**G**), a summary of **P**-values, and the partial eta squared (**R²**) coefficient of determination.

**Bacterial communities**	**Multiple comparison within R1, R2 and R3**									**Correlations among individual bacteria**		
	**R1**			**R2**			**R3**			**R1**	**R2**	**R3**
	**G**	**P**	**R^2**	**G**	**P**	**R^2**	**G**	**P**	**R^2**	**Grouping**		
Rhizobiales	A	****	0.9151	A	***	0.9997	A	****	0.9998	A	A	B
Micropepsales	CD	**	0.6163	B	**	0.9854	E	ns	0.8602	A	A	B
Chitinophagales	E	**	0.9814	B	**	0.9856	CD	**	0.9878	A	A	A
Xanthomodales	E	*	0.7600	C	*	0.9640	E	ns	0.7425	A	A	A
Gemmatimonadales	D	ns	0.9075	C	*	0.9767	E	ns	0.8614	A	A	A
Caulobacterales	B	**	0.5520	B	*	0.9580	D	**	0.9831	A	A	A
Flavobacterales	E	***	0.4164	A	****	0.9999	A	**	0.9968	A	A	A
Propionibacteriales	E	ns	0.6003	C	ns	0.6769	D	**	0.9899	B	B	A
Candidatus	CD	ns	0.4616	B	*	0.9678	E	ns	0.8968	B	A	B
Magasanikbacteria	E	***	0.4892	B	*	0.9767	BC	*	0.9266	A	A	B
Bacillales	E	ns	0.9650	C	ns	0.8359	E	***	0.9984	B	B	A
Sphingobacteriales	E	ns	0.7985	B	***	0.9996	E	*	0.9571	B	A	B
Opitutales	C	ns	0.7999	C	ns	0.7825	E	ns	0.8712	A	A	A
Burkholderiales		**	0.5698	B	****	0.9998	B	***	0.9982	A	A	A

Table S2. Statistical analysis of bacterial communities in the rhizosphere (for type). Results of ANOVA with Tukey's Test (α = 0.05) for multiple comparisons, conducted using GraphPad Prism 10.4.0. The experimental conditions are defined as follows: R1 – without tetracycline, R2 – 5 mg/kg tetracycline, R3 – 25mg/kg tetracycline. The table also includes grouping results (**G**), a summary of **P**-values, and the partial eta squared (**R^2**) coefficient of determination.

**Bacterial communities**	**Multiple comparison within R1, R2 and R3**									**Correlations among individual bacteria**		
	**R1**			**R2**			**R3**			**R1**	**R2**	**R3**
	**G**	**P**	**R^2**	**G**	**P**	**R^2**	**G**	**P**	**R^2**	**Grouping**		
Proteobacteria	A	***	0.9994	A	***	0.9986	A	****	1	A	A	A
Bacteroidota	B	***	0.9991	B	***	0.9998	B	****	0.9999	A	AB	A
Actinobacteriota	B	***	0.9997	B C	***	0.9998	C	***	0.9997	A	AB	A
Verrucomicrobiota	D	**	0.9823	C D	**	0.9916	F	*	0.9684	B	A	B
Planctomycetota	D	*	0.9792	D E	**	0.9971	E	**	0.9918	A	A	A
Firmicutes	E	*	0.9797	E	**	0.9946	D	***	0.9988	B	B	A
Myxococcota	F			E	ns	0.8806	G			B	A	B
Patescibacteria	F			B D	**	0.9972	G			B	A	B
Chloroflexi	D E	**	0.9851	F			G			A	B	B
Gemmatimonadota	F			F			F	*	0.9693	B	B	A
Acidobacteriota	E	*	0.9636	F			G			A	B	B
Sparse	C	***	0.9992	B C	***	0.9994	B C	***	0.9997	A	A	A

Table S3. Statistical analysis of bacterial communities in the rhizosphere (for class). Results of ANOVA with Tukey's Test (α = 0.05) for multiple comparisons, conducted using GraphPad Prism 10.4.0. The experimental conditions are defined as follows: R1 – without tetracycline, R2 – 5 mg/kg tetracycline, R3 – 25mg/kg tetracycline. The table also includes grouping results (**G**), a summary of **P**-values, and the partial eta squared (**R²**) coefficient of determination.

**Bacterial communities**	**Multiple comparison within R1, R2 and R3**									**Correlations among individual bacteria**		
	**R1**			**R2**			**R3**			**R1**	**R2**	**R3**
	**G**	**P**	**R^2**	**G**	**P**	**R^2**	**G**	**P**	**R^2**	**Grouping**		
Alphaproteobacteria	A	****	0.9999	A	***	0.9997	A	****	1	A	A	A
Bacteroidota	B	***	0.9994	C	****	1	A B	***	0.9998	A	A	A
Actinobacteria	C	***	0.9997	E	***	0.9980	C	**	0.9948	A	B	B
Verrucomicrobiae	D	***	0.9997	F	**	0.9964	F			A	B	C
Planctomycetes	E	***	0.9997	H			F			A	B	C
Bacilli	F	***	0.9987	H			D	*	0.9437	A	B	B
Polyangia	G	*	0.9035	G	***	0.9989	F			B	A	C
Gammaproteobacteria	C	***	0.9992	D	***	0.9997	B C	***	0.9994	A	A	A
Chloroflexia	H			H			E	**	0.9970	B	B	A
ABY1	H			G	**	0.9978	F			B	A	B
Acidimicrobiia	H			H			E	**	0.9811	A	B	B
Sparse	C	***	0.9984	B	***	0.9994	A	****	0.9999	B	A	A

Table S4. Statistical analysis of bacterial communities in the rhizosphere (for family). Results of ANOVA with Tukey's Test (α = 0.05) for multiple comparisons, conducted using GraphPad Prism 10.4.0. The experimental conditions are defined as follows: R1 – without tetracycline, R2 – 5 mg/kg tetracycline, R3 – 25mg/kg tetracycline. The table also includes grouping results (**G**), a summary of **P**-values, and the partial eta squared (**R^2**) coefficient of determination.

**Bacterial communities**	**Multiple comparison within R1, R2 and R3**									**Correlations among individual bacteria**		
	**R1**			**R2**			R3			**R1**	**R2**	R3
	**G**	**P**	R^2	**G**	**P**	R^2	**G**	**P**	R^2	**Grouping**		
Rhizobiaceae	B	***	0.9989	B	**	0.9811	B	**	0.9961	A	AB	B
Micropepsaceae	BC	***	0.9986	B	**	0.9889	C	ns	0.7815	A	A	B
Flavobacteriaceae	A	***	0.9994	A	****	1	A	**	0.9952	A	A	A
Chitiniphagaceae	BC	*	0.9792	B	***	0.9988	B	**	0.9958	A	A	A
Devosiaceae	BC	**	0.9925	B	**	0.9921	B	**	0.993	A	A	A
Comamonadaceae	C	***	0.9991	C	ns	0.7169	B	*	0.9728	A	B	A
Sphinhobacteriaceae	D	*	0.9156	B	**	0.9944	C	*	0.9143	B	A	B
Planococcaceae	DE	ns	0.8023	C	ns	0.7759	B	**	0.9897	B	B	A
Nocardioidaceae	C	***	0.9991	B	****	0.9998	B	*	0.9783	A	B	A
Gemmatimonadaceae	D	ns	0.7020	B	*	0.9650	B	**	0.9911	B	A	A
Xanthobacteraceae	BC	**	0.9931	C	ns	0.6879	B	**	0.9957	A	B	A
Comamonadaceae	C	**	0.9908	C	*	0.9288	C	ns	0.7973	A	B	B
Caulobacteriaceae	E	ns	0.8178	B	*	0.9565	B	*	0.9572	B	A	A

Table S5. Statistical analysis of bacterial communities in the soil (for class). Results of ANOVA with Tukey's Test (α = 0.05) for multiple comparisons, conducted using GraphPad Prism 10.4.0. The experimental conditions are defined as follows: R1 –without tetracycline, R2 – 5 mg/kg tetracycline, R3 – 25mg/kg tetracycline. The table also includes grouping results (**G**), a summary of **P**-values, and the partial eta squared (**R²**) coefficient of determination.

**Bacterial communities**	**Multiple comparison within R1, R2 and R3**									**Correlations among individual bacteria**		
	**R1**			**R2**			**R3**			**R1**	**R2**	**R3**
	**G**	**P**	**R^2**	**G**	**P**	**R^2**	**G**	**P**	**R^2**	**Grouping**		
Alphaproteobacteria	A	****	1	C	****	1	C	****	0.9999	A	B	B
Bacteroidota	B	****	1	E	****	0.9987	E	***	0.9983	A	B	B
Actinobacteria	D	**	0.9966	B	***	0.9999	B	****	1	AB	A	A
Verrucomicrobiae	D	***	0.9998	F	****	0.9999	G			A	B	B
Planctomycetes	E	**	0.9923	G			F	**	0.9819	A	A	A
Bacilli	E	**	0.9896	C	****	0.9998	B C	***	0.9997	B	A	A
Polyangia	E	**	0.9952	G			G			A	B	B
Gammaproteobacteria	C	***	0.9984	D	***	0.9997	D	***	0.9993	A	A	A
Chloroflexi	F			E	***	0.9989	E	**	0.9927	B	A	A
Gemmatimonadetes	F			F	**	0.9923	F	***	0.9982	B	A	A
Sparse	B C	****	0.9999	A	****	0.9999	A	****	0.9999	B	A	A

Table S6. Statistical analysis of bacterial communities in the soil (for type). Results of ANOVA with Tukey's Test (α = 0.05) for multiple comparisons, conducted using GraphPad Prism 10.4.0. The experimental conditions are defined as follows: R1 – without tetracycline, R2 – 5 mg/kg tetracycline, R3 – 25mg/kg tetracycline. The table also includes grouping results (**G**), a summary of **P**-values, and the partial eta squared (**R^2**) coefficient of determination.

**Bacterial communities**	**Multiple comparison within R1, R2 and R3**									**Correlations among individual bacteria**		
	**R1**			**R2**			**R3**			**R1**	**R2**	**R3**
	**G**	**P**	**R^2**	**G**	**P**	**R^2**	**G**	**P**	**R^2**	**Grouping**		
Proteobacteria	A	****	0.9999	A	***	0.9994	A	***	0.9998	A	B	B
Bacteroidota	B	***	0.9990	D	**	0.9838	D	**	0.9805	A	B	B
Actinobacteriota	C	****	1	A	***	0.9996	A	***	0.9994	B	A	A
Verrucomicrobiota	C	***	0.9984	F			F			A	B	B
Planctomycetota	D	*	0.9792	D	**	0.9971	D	**	0.9918	A	A	A
Firmicutes	D	*	0.9464	B	**	0.9942	B	****	1	B	A	A
Myxococcota	D	***	0.9995	F			F			A	B	B
Patescibacteria	D	*	0.9765	F			F			A	B	B
Chloroflexi	E			B C	***	0.9994	B C	**	0.9863	B	A	A
Gemmatimonadota	E			D	***	0.9994	D	***	0.9995	B	A	A
Acidobacteriota	E			E	**	0.9868	E	**	0.9938	B	A	A
Sparse	C	***	0.9992	C	****	0.9999	C	****	0.9999	A	A	A

Table S7. Statistical analysis of bacterial communities in the soil (for order). Results of ANOVA with Tukey's Test (α = 0.05) for multiple comparisons, conducted using GraphPad Prism 10.4.0. The experimental conditions are defined as follows: R1 – without tetracycline, R2 – 5 mg/kg tetracycline, R3 – 25mg/kg tetracycline. The table also includes grouping results (**G**), a summary of **P**-values, and the partial eta squared (**R^2**) coefficient of determination.

**Bacterial communities**	**Multiple comparison within R1, R2 and R3**									**Correlations among individual bacteria**		
	**R1**			**R2**			**R3**			**R1**	**R2**	**R3**
	**G**	**P**	**R^2**	**G**	**P**	**R^2**	**G**	**P**	**R^2**	**Grouping**		
Rhizobiales	A	***	0.9995	A B	***	0.9991	B	***	0.9988	A	A	B
Micropepsales	B	***	0.9991	E	ns	0.8977	B	***	0.9989	A	B	A
Burkholderiales	B	**	0.9974	C D	**	0.9948	C D	**	0.9851	A	A	A
Flavobacteriales	B	**	0.9973	E	*	0.9384	E	ns	0.7499	A	B	B
Chitinophagales	B C	***	0.9986	E	ns	0.7651	E	ns	0.6656	A	B	B
Opitutales	C D	**	0.9965	E	ns	0.7381	E	*	0.9108	A	B	B
Sphingobacteriales	D	*	0.9447	E	*	0.9791	E	*	0.9237	A	B	B
Xanthomodales	D	*	0.9789	E	**	0.9844	E	ns	0.8937	A	B	B
Bacillales	E	ns	0.8715	A	****	0.9999	A	***	0.9998	B	A	A
Micrococcales	E	ns	0.8715	B	***	0.9992	B	***	0.999	B	A	A
Gemmatimonadales	E	ns	0.8778	C D	***	0.9991	D	***	0.9997	B	A	A
Thermomicrobiales	E	ns	0.8005	B C	**	0.9897	B C	**	0.9979	B	A	A
Propionibacteriales	E	*	0.9657	E	ns	0.6562	C D	**	0.9973	B	B	A
Giit GS	E	*	0.9084	D	*	0.918	E	ns	0.8524	B	A	B

Table S8. Statistical analysis of bacterial communities in the soil (for family). Results of ANOVA with Tukey's Test (α = 0.05) for multiple comparisons, conducted using GraphPad Prism 10.4.0. The experimental conditions are defined as follows: R1 – without tetracycline, R2 – 5 mg/kg tetracycline, R3 – 25mg/kg tetracycline. The table also includes grouping results (**G**), a summary of **P**-values, and the partial eta squared (**R²**) coefficient of determination.

**Bacterial communities**	**Multiple comparison within R1, R2 and R3**									**Correlations among individual bacteria**		
	**R1**			**R2**			**R3**			**R1**	**R2**	**R3**
	**G**	**P**	**R^2**	**G**	**P**	**R^2**	**G**	**P**	**R^2**	**Grouping**		
Rhizobiaceae	BC	**	0.9968	CD	**	0.9877	E	ns	0.8196	A	B	C
Micropepsaceae	A	***	0.9991	E	*	0.9284	E	ns	0.8636	A	B	B
Flavobacteriaceae	AB	**	0.9903	E	*	0.9658	E	*	0.9614	A	B	B
Chitiniphagaceae	BC	****	0.9999	E	*	0.9494	E	ns	0.9017	A	B	B
Devosiaceae	C	**	0.9845	E	*	0.9748	E	*	0.9664	A	B	B
Comamonadaceae	C	**	0.9975	E	ns	0.8947	E	*	0.9109	A	B	B
Opitutaceae	C	***	0.9991	E	*	0.9050	E	ns	0.8788	A	B	B
Sphinobacteriaceae	C	***	0.9992	E	*	0.9205	E	*	0.9657	A	B	B
Planococcaceae	D	ns	0.8712	A	**	0.995	A	**	0.9957	B	A	A
Bacillaceae	D	*	0.9030	AB	***	0.9988	A B	***	0.9990	B	A	A
Nocardioidaceae	D	ns	0.8868	BC	**	0.9947	B C	****	0.9999	B	A	A
Gemmatimonadaceae	D	ns	0.8414	BC	**	0.9874	C D	**	0.9970	B	A	A
Gitt - GS	D	ns	0.8525	CD	*	0.9763	C D	**	0.9830	B	A	A
Micrococcaceae	D	*	0.9788	D	***	0.9996	D	**	0.9884	B	A	A

Table S9. Calculations of the Chi-Square (X^2) test for the order and family of bacteria, including Yates' correction. In the calculations, it was assumed that the expected value consists of microorganisms present without the R1 antibiotic, while the variables represent microorganisms found on roots originating from soil contaminated with tetracycline. The experimental conditions are defined as follows: R1 – without tetracycline, R2 – 5 mg/kg tetracycline, R3 – 25mg/kg tetracycline. The calculations were performed using the Preacher [2001] online program.

	**R1×R2**				**R1×R3**			
	**X^2**	***P* value**	***Yates'* X^2**	***Yates' P* value**	**X^2**	***P* value**	***Yates'* X^2**	***Yates' P* value**
**order**								
Rhizobiales	7.288	0.0263	6.066	0.0481	9.521	0.008	8.106	0.0173
Micropepsales	17.715	0.0001	15.149	0.0005	0.422	0.7946	0.200	0.9048
Burkholderiales	5.564	0.0676	4.211	0.1217	5.080	0.0934	3.845	0.1462
Flavobacteriales	16.312	0.0002	13.744	0.0010	15.754	0.0004	13.238	0.0013
Chitinophagales	11.293	0.0035	8.915	0.0115	11.446	0.0032	9.049	0.0108
Opitutales	7.055	0.0294	4.922	0.0853	8.214	0.0165	5.891	0.0525
Sphingobacteriales	6.017	0.0493	4.025	0.1336	6.366	0.0414	4.285	0.1173
Xanthomodales	6.145	0.0463	4.071	0.1306	5.716	0.0573	3.733	0.1546
Bacillales	307.251	0	275.476	0	474.856	0	435.148	0
Micrococcales	117.704	0	98.400	0	112.840	0	93.964	0
Gemmatimonadales	31.328	0	21.647	0	25.369	0	16.728	0.0002
Thermomicrobiales	69.406	0	54.25	0	82.239	0	65.484	0
Propionibacteriales	0.014	0.9928	0.874	0.6459	40.366	0	28.684	0
Giit GS	17.023	0.0002	10.829	0.0044	0.026	0.9871	0.731	0.6938
**family**								
Rhizobiaceae	2.066	0.3559	1.063	0.5877	9.546	0.0084	7.213	0.0271
Micropepsaceae	18.909	0	16.252	0.0002	17.661	0.0001	15.097	0.0005
Flavobacteriaceae	15.517	0.0004	13.004	0.0015	15.771	0.0003	13.242	0.0013
Chitiniphagaceae	9.858	0.0072	7.483	0.0237	10.272	0.0058	7.845	0.0197
Devosiaceae	8.34	0.01545	6.039	0.0488	7.891	0.0193	5.669	0.0587
Comamonadaceae	7.032	0.0297	4.885	0.0869	7.159	0.0278	4.991	0.0824
Opitutaceae	6.893	0.0318	4.762	0.0924	7.031	0.0297	4.875	0.0873
Sphinobacteriaceae	4.987	0.0826	3.044	0.2182	5.429	0.0662	3.388	0.1837
Planococcaceae	113.132	0	92.534	0	182.433	0	156.029	0
Bacillaceae	47.496	0	34.944	0	77.178	0	60.919	0
Nocardioidaceae	40.151	0	28.349	0	42.28	0	30.092	0
Gemmatimonadaceae	42.695	0	30.24	0	35.362	0	23.974	0
Gitt - GS	16.061	0.0003	9.645	0.0080	16.666	0.0002	9.985	0.0067
Micrococcaceae	14.283	0.0007	7.612	0.0222	14.380	0.0007	7.731	0.0209
